# Feasibility of the ACTPlan Program for African American Dementia Caregivers: A Self-Directed Multimedia Delivery

**DOI:** 10.1093/geroni/igab046.584

**Published:** 2021-12-17

**Authors:** Yashika Watkins, William Collinge, Alysha Hart, Rita Tharpe, Neelum Aggarwal, Gloria Bonner

**Affiliations:** 1 Chicago State University, Cliffside Park, New Jersey, United States; 2 Collinge and Associates, Inc., Eugene, Oregon, United States; 3 University of Illinois at Chicago College of Nursing, Chiavgo, Illinois, United States; 4 University of Illinois at Chicago College of Nursing, Chicago, Illinois, United States; 5 Rush University, Chicago, Illinois, United States; 6 Unversity of Illinois at Chicago College of Nursing, Chicago, Illinois, United States

## Abstract

African Americans (AA) are less likely than White Americans to complete advance care plans or end-of-life treatment documents. They face significantly greater risk of Alzheimer’s Disease, a silent epidemic for this population, and other dementias. The healthcare system’s lack of dementia support for AAs contributes to disparate care. A four-session caregiver group education program was conducted on advance care planning for AA dementia family providers. The program was based on Kolb’s Experiential Learning Model and initially found effective in an R01 study using in-person delivery by a professional. The present pilot assessed feasibility of delivering the program in a self-directed multimedia format without professional facilitation, using Session 1 on tube feeding decisions as the test session. Twenty-six AA dementia caregivers completed the session in groups of 5 to 8 at a church equipped with a large TV screen. On-screen prompts guided navigation through the program which included recorded lecture, slides, short videos on decision-making, and group discussions. Using quantitative and qualitative methods, pre-and post-survey instruments were administered and interviews conducted. Usability ratings averaged 84%. Knowledge and self-efficacy gains exceeded those of the R01, with a 35% increase in correct responses on knowledge items, versus 18% for the R01 subjects; and increase in perceived decisional self-efficacy of 31% versus 30% for the R01 subjects. Qualitative feedback was universally positive. These findings confirm the feasibility of the self-guided multimedia approach to delivery of the program. A large RCT is planned which, if successful, will support wide dissemination to AA caregivers in need.

